# An HIV-1 capsid binding protein TRIM11 accelerates viral uncoating

**DOI:** 10.1186/s12977-016-0306-5

**Published:** 2016-10-13

**Authors:** Ting Yuan, Weitong Yao, Kenzo Tokunaga, Rongge Yang, Binlian Sun

**Affiliations:** 1 Research Group of HIV Molecular Epidemiology and Virology, Center for Emerging Infectious Diseases, The State Key Laboratory of Virology, Wuhan Institute of Virology, Chinese Academy of Sciences, Xiaohongshan 44, Wuhan, 430071 People’s Republic of China; 2 Department of Pathology, National Institute of Infectious Diseases, Tokyo, Japan

**Keywords:** TRIM11, HIV-1, Uncoating, Capsid

## Abstract

**Background:**

Several members of the TRIM family have been implicated in antiviral defense. Our previous report showed that human TRIM11 potently inhibited HIV-1 transduction by reducing the viral reverse transcripts. These results prompted us to examine the effect of TRIM11 on HIV-1 uncoating, which is closely related to viral reverse transcription.

**Results:**

Using a combination of in vitro binding and in situ proximity ligation assay, we showed that TRIM11 could interact with HIV-1 capsid. Overexpression of TRIM11 accelerates HIV-1 uncoating and reduces viral reverse transcription indicated by the fate-of-capsid assay and quantitative PCR respectively. Knockdown of TRIM11 enhanced HIV-1 capsid stability and increased viral reverse transcription. However, the replication of another retrovirus MLV is not affected by TRIM11. Moreover, the reverse transcription of HIV-1 mutant bearing capsid G89V showed insensitivity to restriction by TRIM11, indicating that the viral determinant of restriction by TRIM11 might reside on capsid. Using microtubule dynamics inhibitors, we revealed that microtubule dynamics contributes to TRIM11-mediated HIV-1 capsid premature disassembly and the reduction of reverse transcription levels. Finally, we demonstrated that TRIM11 inhibits HIV-1 transduction and accelerates viral uncoating in HIV-1 permissive THP-1-derived macrophages.

**Conclusions:**

We identify TRIM11 as a new HIV-1 capsid binding protein. Our data also reveal that TRIM11 restricts HIV-1 reverse transcription by accelerating viral uncoating, and microtubule dynamics is implicated in TRIM11-imposed block to early events of HIV-1 replication.

**Electronic supplementary material:**

The online version of this article (doi:10.1186/s12977-016-0306-5) contains supplementary material, which is available to authorized users.

## Background

The human tripartite motif containing (TRIM) protein family includes approximately 100 members. These proteins have diverse functions ranging from DNA damage signaling to antiviral activities. The TRIM family is characterized by its conserved tripartite motif/RBCC motif on the N-terminal, which consists of RING, B-Box and C-coiled domains. The variable C-terminal is responsible for protein diversity. Of the TRIM family proteins which have been demonstrated possessing antiviral activities, TRIM5α is the best characterized one, which has been identified as a potent restriction factor against retroviruses in a species-specific manner [1, 2]. Rhesus monkey TRIM5α (TRIM5α_rh_) restricts HIV-1 replication via multiple mechanisms [1, 3–6]: (1) TRIM5α_rh_ recognizes HIV-1 capsid causing premature uncoating and reduced viral reverse transcription [1, 3]. (2) TRIM5α_rh_ blocks viral DNA nuclear import [4, 5], and (3) it induces rapid degradation of HIV-1 gag polyproteins that cause reduction of virion release in late stage of viral replication [6]. In contrast, human TRIM5α (TRIM5α_hu_) only weakly inhibits HIV-1 as it does not recognize the viral capsid [3, 7].

After entry into a target cell, the HIV-1 capsid, composed of hexamers and pentamers, undergoes dissociation from the viral complex, a process known as uncoating [8]. However, increasing evidences challenge the traditional notion that the core completely disassembles soon after viral entry by proposing the model of cytoplasmic uncoating or uncoating at the nuclear pores [9, 10]. Meanwhile, the HIV-1 RNA genome is reverse transcribed into double stranded DNA, which constitutes pre-integration complex with multiple other proteins like Vpr and Integrase. Increasing evidences suggest that there are multiple interplays between viral uncoating and other early steps of HIV-1 replication cycle. The abnormal viral capsid uncoating often causes impairment in HIV-1 replication. HIV-1 premature uncoating induced by TRIM5α_rh_ and owl monkey TRIMCyp has been shown to reduce reverse transcription [3, 11]. Dochi et al. reported that the lack of phosphorylation of capsid residues Ser16-Pro17, either by mutation or by suppression of kinase ERK2 packaging, can impair capsid recognition by a peptidyl-prolyl isomerase Pin1, ultimately reducing reverse transcription and causing viral core to accumulate in the cytoplasm [12]. They hypothesize that a molecular switch is needed to promote uncoating and reverse transcription [12]. It has been shown that reverse transcription inhibitor not only delay uncoating but also increase the stability of viral core in the presence of restriction factors like TRIM5α_rh_ [13, 14]. These results suggest that reverse transcription process within the core has the ability to impact the kinetics of uncoating. Moreover, mutation in capsid protein that enhanced binding to a cytopasmic protein, cyclophilin A (CypA), enhanced capsid stability and impeded viral DNA nuclear import [15]. MxB has recently been identified as a restriction factor inhibiting viral DNA entry into nucleus and stabilizing the viral capsid [16]. These results suggest that abnormal stability of viral capsid may impede nuclear entry of viral DNA. All together, these findings indicate that HIV-1 uncoating is a fine process which is regulated by multiple cellular and viral proteins, and is a vulnerable target of restriction factors.

A screen of human and mouse TRIM family members identified TRIM11 as an inhibitor of both early and late stages of HIV-1 replication [17]. We recently reported that TRIM11 restricts the early stage of HIV-1 replication by reducing viral reverse transcription, and that TRIM11 inhibits the LTR activity of HIV-1 in an NF-κB dependent way [18]. Accordingly, Lee et al. reported that TRIM11 is a negative regulator of RIG-I-mediated NF-κB activity [19]. However, the precise mechanism by which TRIM11 inhibits viral reverse transcription is not well characterized. The influence of uncoating on reverse transcription prompted us to explore whether TRIM11 could recognize HIV-1 capsid and alter viral capsid stability at early stage of postinfection.

## Results

### TRIM11 interacts with HIV-1 capsid

**Fig. 1:**
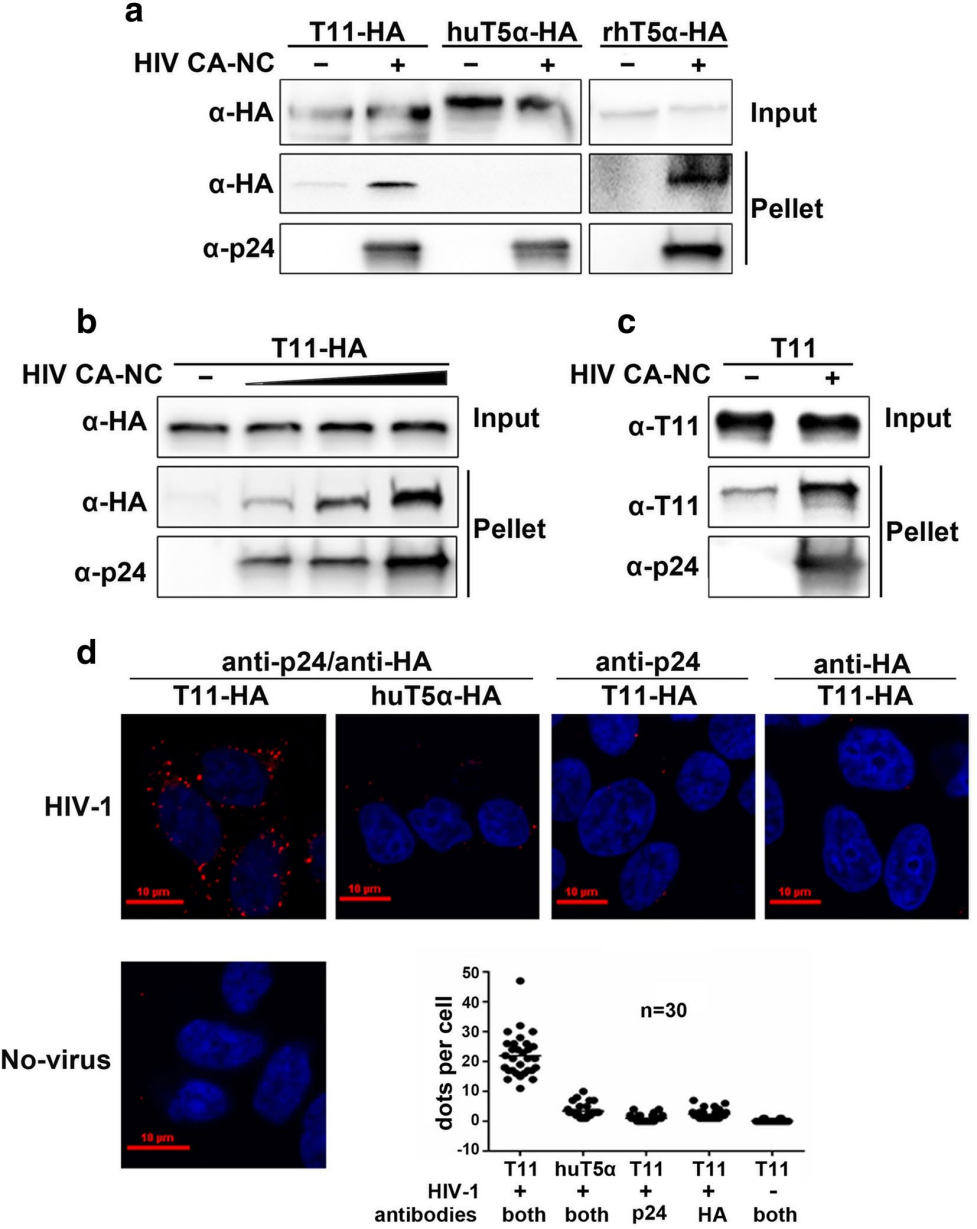
TRIM11 binds to HIV-1 capsid. **a** The in vitro assembled HIV-1 CA-NC protein was incubated with HA-antibody precipitated mixture from HEK293 cell lysates containing the indicated TRIM proteins, and an aliquot of the mixture was retained as input. After centrifugation through 70 % sucrose cushion, the pellet was resuspended in SDS sample buffer. The input and pellet were analyzed by Western blotting with an anti-HA antibody (to detect TRIM proteins) and an anti-p24 antibody (to detect CA-NC). **b** Similar experiment was carried out as described above with HEK293 cell lysates containing TRIM11-HA incubated with increasing amounts of in vitro assembled CA-NC. **c** Similar experiments were carried out as described above with TRIM11 purified from *E. Coli.* incubated with in vitro assembled CA-NC. **d** Cells expressing TRIM11-HA or TRIM5α_hu_-HA were infected with or without HIV-1. Four hours post infection, cells were fixed and applied to PLA assay. Nuclei were stained with DAPI (*blue*), and PLA was performed for the association between p24 and TRIM11 (*red*). Quantification of PLA signal was measured by the average spots per cell of 30 cells in each sample. Representative results from three separate experiments are shown

We previously reported that TRIM11 reduced HIV-1 reverse transcription and suppressed viral transduction [18]. Consideration the close relationship between reverse transcription and uncoating described above, we hypothesized that TRIM11 might interact with HIV-1 capsid and accelerate uncoating process. To investigate this possibility, we examined the ability of TRIM11 to bind in vitro assembled HIV-1 capsid-nucleocapsid (CA-NC) complexes, which simulate the surface of viral core and represent an established model for the assessment of HIV-1 capsid interacting proteins [3]. After incubation of HIV-1 CA-NC complexes with cell lysates bearing TRIM5α_rh_-HA or HA-precipitated cell lysates containing TRIM11-HA and TRIM5α_hu_-HA, we deposited the mixtures onto 70 % sucrose cushions and ultracentrifuged them for one hour. We found that both TRIM11 and TRIM5α_rh_ formed complexes with HIV-1 CA-NC complexes, indicated by the appearance of these proteins in the pellet with CA-NC complexes but not in the negative control (Fig. 1a). In contrast, TRIM5α_hu_ did not bind to HIV CA-NC complexes (Fig. 1a), as previously reported [3]. To confirm the interaction between TRIM11 and CA-NC complexes, we incubated TRIM11-HA expressing cell lysates with an increasing concentration of HIV-1 CA-NC complexes, and then ultracentrifuged the mixtures on sucrose cushions. We found that the amount of TRIM11 in the pellet increased with the amount of HIV-1 CA-NC complexes (Fig. 1b). These findings indicated that TRIM11 has the ability to associate with in vitro assembled HIV-1 CA-NC complexes.

Cell lysates contain multiple proteins that interact with HIV-1 capsid. Although TRIM11-HA is abundant, chances are that TRIM11 could pellet with CA-NC complexes by interacting with other HIV-1 capsid-interacting proteins. To rule out this possibility, we incubated purified TRIM11 expressed in *E. coli* with in vitro assembled HIV-1 CA-NC complexes. Clearly, more TRIM11 was pelleted in the presence of CA-NC complexes, even though it could migrate through 70 % sucrose cushions without viral capsid (Fig. 1c). This unexpected pelletable TRIM11 could be caused by the high purity and concentration used in this in vitro system, which may form high-order oligomers. These results suggest that the TRIM11 could interact with HIV-1 CA-NC complexes without other cellular proteins.

To investigate whether TRIM11 could interact with HIV-1 capsid during virus infection, we introduced the proximity ligation assay (PLA) system that detects protein–protein interactions closer than 40 nm in situ. HEK293 cells expressing TRIM11-HA or TRIM5α_hu_-HA were infected with HIV-1 or not for 4 h. The cells were then fixed and incubated with anti-p24 mAbs and anti-HA rAbs simultaneously followed by PLA probes incubation, ligation and amplification. The negative control were incubated with either anti-p24 or anti-HA antibody. The interaction events between TRIM11 and p24 were revealed as bright fluorescent spots, which resulted in an average of 22 spots per cell in TRIM11-HA expressing cells while 3 per cell in TRIM5α_hu_-HA expressing cells (Fig. 1d). The negative controls including only one antibody incubation and TRIM11-HA expressing cells not infected with HIV-1 showed similar dots per cell with TRIM5α_hu_-HA expressing cells infected with HIV-1 (Fig. 1d). These results indicate that TRIM11 associates with HIV-1 capsid during virus infection.

### TRIM11 accelerates HIV-1 uncoating during infection

**Fig. 2:**
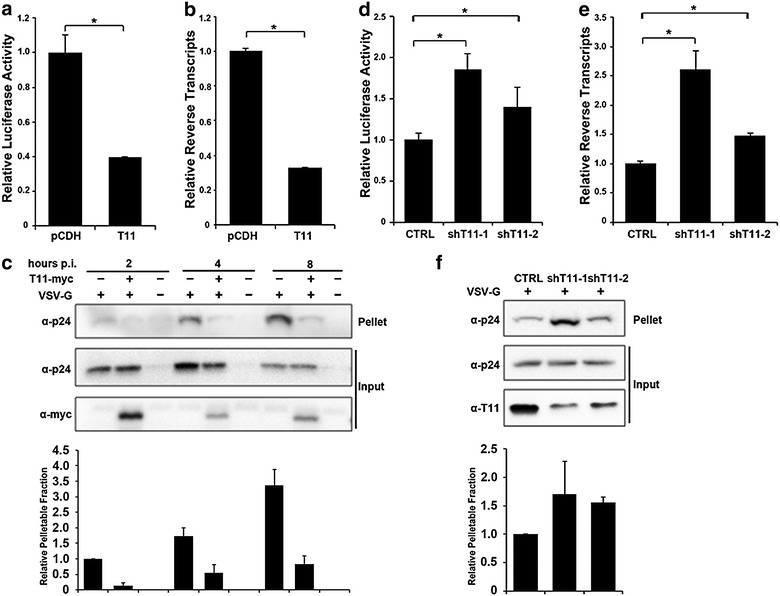
TRIM11 accelerates HIV-1 uncoating during infection. **a**, **b** HEK293 cells stably expressing TRIM11-HA and pCDH were infected with 50 ng/ml (p24^gag^) HIV-1 and viral transduction was assessed at 24 h post infection by luciferase activity (**a**) and late reverse transcription levels were assessed at 3 h post infection by qPCR (**b**). **c** HEK293 cells transfected with pCMV-myc-TRIM11 or empty vector were infected with HIV-1 with or without VSV-G envelop at 4 °C for 30 min and then incubated at 37 °C for the indicated time. The cell lysates were centrifuged through 50 % sucrose cushion, and the pellet was resuspended in SDS sample buffer. The input and pellet were analyzed by Western blotting with an anti-myc and an anti-p24 antibody. The levels of p24 in pelletable and input fractions were measured by densitometry and pellet/input ratio was calculated. **d**–**f** Similar experiments were carried out as described in **a**–**c**, with HEK293 cells in which TRIM11 had been stably knocked down with shRNA. *Error bars* represent the standard deviations from three independent replicates of the same experiments. *P < 0.05

As previously reported [18], single round infectivity assay and quantitative PCR indicated that TRIM11 overexpression significantly inhibited HIV-1 transduction and reverse transcription, in comparison to vector control (Fig. 2a, b). Since TRIM11 associates with HIV-1 capsid during virus infection, we hypothesized that TRIM11 might induce premature uncoating once it recognizes viral capsid. To test this hypothesis, we challenged TRIM11 overexpressing HEK293 cells with HIV-1 for various time and performed the fate-of-capsid assay. We found that pelletable capsid levels were significantly lower in TRIM11 overexpressing cells than that in vector control cells since 2 h post infection (Fig. 2c). Furthermore, we used shRNA to knockdown TRIM11. As expected, knocking down endogenous TRIM11 increased HIV-1 infectivity (Fig. 2d) as well as viral reverse transcription (Fig. 2e), and significantly enhanced HIV-1 capsid stability (Fig. 2f). These results suggest that TRIM11 accelerates HIV-1 uncoating and decreases reverse transcription levels during virus infection.

**Fig. 3:**
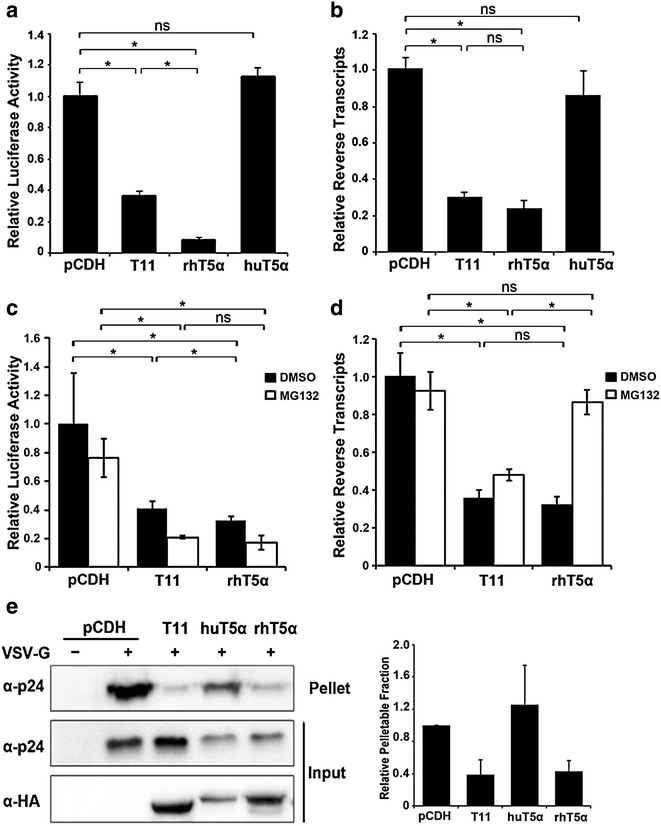
Comparison of the effects of different TRIM proteins on the early stages of HIV-1 replication. **a**, **b** The indicated HEK293 cell lines were infected with 50 ng/ml (p24^gag^) HIV-1 and viral transduction was analyzed by luciferase activity (**a**) and late reverse transcripts were measured at 24 h post infection by qPCR (**b**). **c** The indicated HEK293 cell lines were infected with HIV-1 for 3 h. Cell lysates were processed for the fate-of-capsid assay as described in Fig. 2c. The levels of p24 in pelletable and input fractions were measured by densitometry and pellet/input ratio was calculated. *Error bars* represent the standard deviations from three independent replicates. *P < 0.05

We found that TRIM11 and HIV-1 restriction factor TRIM5α_rh_ share the capacity to bind HIV-1 CA-NC complexes and to accelerate uncoating. Although TRIM11 and TRIM5α belong to the same subfamily, a phylogenetic analysis reveals that their amino acid sequences are quite different [20]. In addition, TRIM5α_hu_ mediates weak inhibition of the early stage of HIV-1 replication because it fails to recognize the viral capsid [3, 7, 21]. To discriminate between the effects of the three TRIM proteins on HIV-1 infection, we compared HIV-1 transduction levels in cells overexpressing TRIM11, TRIM5α_rh_ and TRIM5α_hu_. The results showed that HIV-1 transduction was less restricted by TRIM11 than TRIM5α_rh_, whereas it was not affected by TRIM5α_hu_ (Fig. 3a). Interestingly, HIV-1 late reverse transcription was reduced to a similar level in both TRIM5α_rh_ and TRIM11 overexpressing cells (Fig. 3b). It is reported that TRIM5α_rh_ could inhibit not only reverse transcription by accelerating viral capsid uncoating, but also nuclear accumulation of viral DNA [4, 5]. Our previous study suggested that TRIM11 does not impede viral DNA nuclear import [18], which would explain the relatively moderate restriction of TRIM11 on HIV-1 transduction (Fig. 3a). Previous studies showed that TRIM5α_rh_ restriction of HIV-1 reverse transcription could be restored by proteasome inhibitors, despite viral transduction remained blocked [4, 5]. In order to see if this is the case for TRIM11, we examined HIV-1 transduction and reverse transcription levels impeded by both TRIM11 and TRIM5α_rh_ in the present of proteasome inhibitor MG132. In contrast to TRIM5α_rh_, the restriction of TRIM11 on HIV-1 transduction and reverse transcription were insensitive to MG132 (Fig. 3c, d), suggesting a different mechanism by which TRIM11 restricts early stages of HIV-1 replication compared to TRIM5α_rh_. The results of an in vivo uncoating assay also suggested that both TRIM11 and TRIM5α_rh_ cause a premature capsid disassembly (Fig. 3e), whereas TRIM5α_hu_ did not influence the stability of HIV-1 capsid. Collectively, both TRIM11 and TRIM5α_rh_ inhibit HIV-1 reverse transcription at a comparable level by causing premature viral capsid disassembly.

### Microtubule dynamics contributes to TRIM11-mediated premature disassembly of HIV-1 capsid

**Fig. 4:**
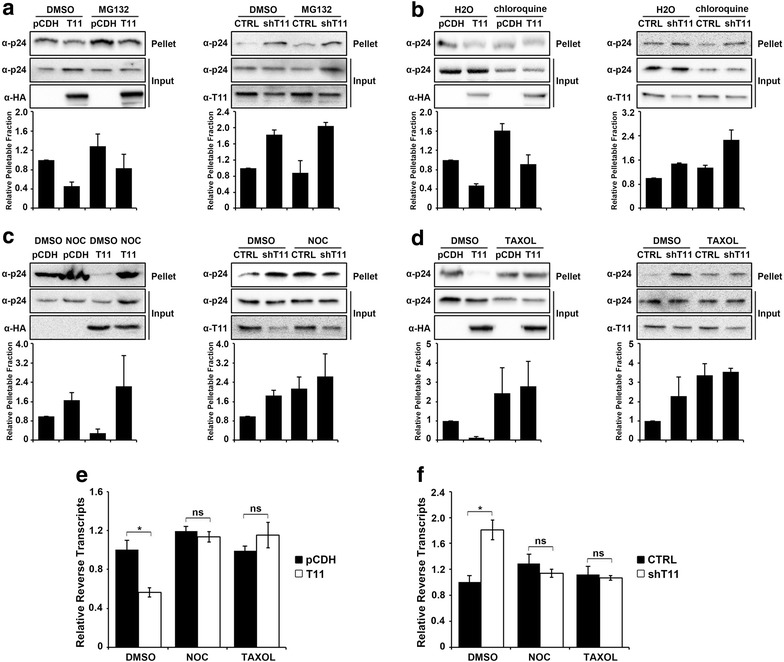
Disruption of microtubule dynamics aborts TRIM11-mediated HIV-1 premature disassembly and reduction of reverse transcription levels. **a**, **b** The indicated HEK293 cell lines were treated with DMSO, 10 μM MG132 (**a**) or 10 μM CQ (**b**) for 3 h before infection with HIV-1. Three hours post infection, cell lysates were processed for the fate-of-capsid assay as described in Fig. 2c. **c**, **d** The indicated cell lines were infected with HIV-1 in the presence of DMSO, 0.1 μM nocodazole (**c**) and 0.1 μM taxol (**d**) for 3 h. Cell lysates were processed for the-fate-of capsid assay as described in Fig. 2c. **e**, **f** HEK293 cells expressing TRIM11-HA and vector (**e**), HEK293 cells stably knocked down TRIM11 (**f**) were infected with 50 ng/ml (p24^gag^) HIV-1 in the presence of DMSO, 0.1 μM nocodazole and 0.1 μM taxol and analyzed by qPCR for late reverse transcripts at 3 h post infection. *Error bars* represent the standard deviations from three independent replicates. *P < 0.05

TRIM5α_rh_-mediated restriction of HIV-1 reverse transcription is rescued by proteasome inhibitors [4, 5], while TRIM11 is not (Fig. 3d). We therefore examined whether TRIM11 accelerates HIV-1 uncoating through the proteasomal or lysosomal pathway. Cells bearing TRIM11-HA or control vector were treated with MG132 (proteasomal inhibitor) or chloroquine (lysosomal inhibitor) followed by HIV-1 infection. Neither MG132 nor chloroquine treatment rescued p24 protein levels in the pellet of TRIM11 overexpressing cells to that observed in control cells (Fig. 4a, b). Also in TRIM11 knockdown cells, the increased pelletable capsid levels were not influenced by either MG132 or chloroquine (Fig. 4a, b). These results indicate that neither proteasome activity nor lysosomal acidification is required for TRIM11-mediated accelerating of HIV-1 uncoating.

Microtubule dynamics has been implicated in both HIV-1 uncoating [22] and TRIM5α_rh_-mediated virus restriction [23]. In order to address whether microtubule dynamics is involved in TRIM11-mediated premature uncoating, we introduced the inhibitors nocodazole and taxol, which prevents microtubule polymerization and disassembly respectively. We found that compared to MG132 and chloroquine, nocodazole treatment resulted in similar pelletable capsid levels between TRIM11 overexpressing cells and control cells (Fig. 4c). As shown in Fig. 4d, taxol treatment increased pelletable capsid in TRIM11-HA expressing cells to the similar level with control cells (Fig. 4d), suggesting that the microtubule dynamics disrupted by nocodazole and taxol could fully restore core stability in TRIM11 overexpressing cells. Meanwhile, both nocodazole and taxol treatment abolished the induction effect of TRIM11 knockdown on HIV-1 capsid stability (Fig. 4c, d). As previous reported [22, 23], microtubule dynamics disruption imposed by nocodazole and taxol treatment could stabilize HIV-1 capsid during early stage of infection. Here we represented that the effects of microtubule dynamics inhibitors on HIV-1 capsid are not compromised by TRIM11 knockdown, suggesting that TRIM11 does not contribute to this effect of microtubule dynamics on HIV-1 replication (Fig. 4c, d).

In accordance with the fate-of-capsid assay, treatment with both nocodazole and taxol for 3 h fully rescued HIV-1 reverse transcription in TRIM11 overexpressing cells (Fig. 4e). Furthermore, both nocodazole and taxol treatment abolished the induction effect of TRIM11 knockdown on viral reverse transcription (Fig. 4f). These results suggest that the effect of TRIM11 on HIV-1 capsid stability and viral reverse transcription levels is dependent on microtubule dynamics.

### MLV and HIV-1 capsid mutant G89V is insensitive to restriction by TRIM11 on early stage of replication

**Fig. 5:**
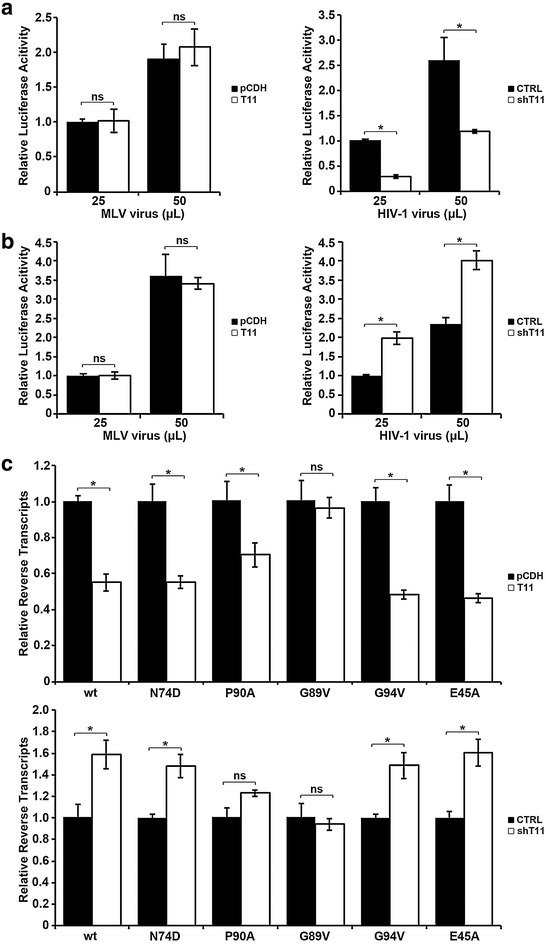
MLV and HIV-1 bearing capsid G89V mutation are insensitive to restriction by TRIM11 on the early stage of replication. **a** HEK293 cells expressing TRIM11-HA or empty vector pCDH were infected with different amount of HIV-1 or MLV, and viral transduction was assessed at 24 or 48 h post infection by luciferase activity. **b** Similar experiments were carried out as described in **a**, with HEK293 cells in which TRIM11 expression levels were stably knocked down with shRNA-1 specific to TRIM11. **c** The indicated HEK293 cell lines were infected with 20 ng/ml (p24^gag^) wild type HIV-1 as well as different capsid mutants and late reverse transcription levels were assessed at 6 h post infection by qPCR. *Error bars* represent the standard deviations from three independent replicates. *P < 0.05

To assess whether the restriction of TRIM11 on HIV-1 was specific, we tested the effect of TRIM11 on another retrovirus, NB tropic mouse leukemia virus (MLV). TRIM11-HA expressing cells and control cells were inoculated with different amount of MLV or HIV-1. Fourty-eight hours later, cells lysis were applied to luciferase assay to determine viral transduction levels. As shown in Fig. 5a, MLV transduction levels were not influenced by TRIM11 overexpression while HIV-1 transduction levels were significantly compromised. In accordance with these results, TRIM11 knockdown potentiated HIV-1 transduction while did not affect MLV transduction (Fig. 5b), suggesting that TRIM11 does not inhibit MLV transduction as it does to HIV-1.

The results presented above indicated that TRIM11 recognizes HIV-1 capsid during infection and accelerates viral uncoating, resulting in reduced reverse transcription. To demonstrate that HIV-1 capsid is the target of TRIM11, we introduced several capsid mutations. E45A mutant has been reported to increase the capsid stability [24], N74D resides in a conserved hydrophobic binding pocket [25], while P90A, G89V and G94V reside in the CypA-binding loop [26]. The reverse transcription levels of HIV-1 bearing these capsid mutations in TRIM11-HA expressing cells and TRIM11 knockdown cells were assessed by realtime PCR. Similar to wild type HIV-1, the reverse transcription levels of E45A and N74D were restricted in TRIM11-HA expressing cells while enhanced in TRIM11 knockdown cells (Fig. 5c), indicating that the effect of TRIM11 on HIV-1 early stages of replication probably does not relate to capsid stability or the hydrophobic binding pocket. In contrast, the effect of TRIM11 on HIV-1 reverse transcription was abolished by capsid mutation G89V, and compromised by capsid mutation P90A (Fig. 5c). Meanwhile, another capsid mutant G94V that also resides on the CypA-binding loop was still sensitive to restriction of TRIM11 (Fig. 5c). Thus, it is premature to conclude that TRIM11 targets the CypA-binding loop of HIV-1 capsid. However, these results strongly suggested that TRIM11 might target viral capsid during early stages of HIV-1 replication.

### TRIM11 interferes with early steps of viral replication in HIV-1 permissive cells

**Fig. 6:**
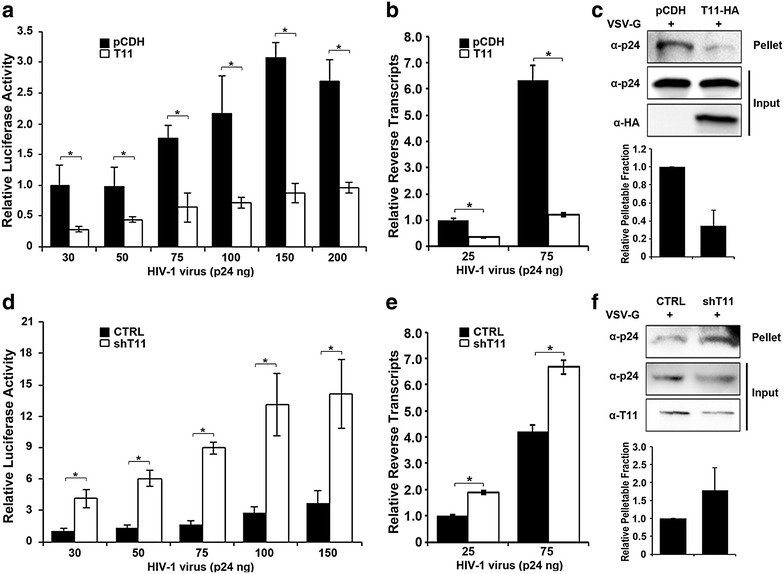
TRIM11 restricts HIV-1 early stage of replication in THP-1 cells. **a**, **b** THP-1 cells expressing TRIM11-HA or empty vector pCDH were treated with 100 nM PMA for 48 h followed by infection with indicated amounts of HIV-1 and viral transduction was analyzed by luciferase activity at 48 h post infection (**a**), and late reverse transcripts were measured by qPCR (**b**). **c** The indicated THP-1 cell lines were treated with 100 nM PMA for 48 h followed by infection with HIV-1 for 1 h. Cell lysates were processed for the fate-of-capsid assay as described in Fig. 2c. The levels of p24 in pelletable and input fractions were measured by densitometry and pellet/input ratio was calculated. **c**–**e** Similar experiments were carried out as described in **a**–**c**, with THP-1 cells in which TRIM11 expression levels were stably knocked down with shRNA-1 specific to TRIM11. *Error bars* represent the standard deviations from three independent replicates. *P < 0.05

To determine the effect of TRIM11 on HIV-1 replication in permissive cells, we first measured the expression of TRIM11 in HEK293, THP-1, THP-1-derived macrophages and Jurkat cells. The results showed that TRIM11 expresses at similar levels in these cells (Additional file 1: Fig. S1). Then we constructed TRIM11 overexpressing and control THP-1 cell lines, which were inoculated with increasing amounts of HIV-1 following PMA treatment. Forty-eight hours post infection, luciferase activity was measured to determine HIV-1 transduction. In contrast to control vectors, TRIM11 overexpression substantially reduced HIV-1 transduction (Fig. 6a) and realtime PCR indicated that TRIM11 overexpression decreased viral reverse transcription levels in THP-1 derived macrophages (Fig. 6b). The fate-of-capsid assay indicated that TRIM11 overexpression reduced the level of assembled viral capsid by about 30 % at 1 h post HIV-1 infection (Fig. 6c). To assess whether physiological levels of TRIM11 in THP-1 cells potentiates HIV-1 replication, we constructed a TRIM11 knockdown THP-1 cell line using the same shRNA as we did in HEK293 cells. In accordance with the results above, knockdown of TRIM11 substantially enhanced HIV-1 transduction regardless of the inoculation concentration (Fig. 6d). Furthermore, knockdown of TRIM11 enhanced HIV-1 reverse transcription levels (Fig. 6e) as well as HIV-1 capsid stability by about 50 % at 1 h post infection (Fig. 6f). Taken together, these results suggest that TRIM11 blocks early steps of HIV-1 replication and accelerates viral uncoating in both THP-1-derived macrophages and HEK293 cells.

## Discussion

TRIM11 is reported to interfere with both early and late stages of viral replication [17, 18]. We previously reported that TRIM11 inhibits HIV-1 reverse transcription during early steps of the viral replication cycle [18]. However, the mechanism by which TRIM11 decreases viral DNA is still unclear. In this study, we demonstrated that TRIM11 both purified from *E. coli.* and expressed in cells interacts with in vitro assembled HIV-1 capsid. Importantly, the PLA assay showed that TRIM11 associates with HIV-1 capsid during viral infection. We then revealed that TRIM11 accelerates HIV-1 uncoating during viral infection in both HEK293 cells and THP-1-derived macrophages. The effect of TRIM11 on uncoating is dependent on microtubule dynamics, whereas independent of proteasomal or lysosomal pathway. All of these findings support the restriction of HIV-1 transduction by TRIM11, while MLV transduction is not influenced by TRIM11. The HIV-1 CA mutant G89V is insensitive to the effect of TRIM11, which strengthened the possibility that the capsid of HIV-1 could be the determinant for restriction by TRIM11. Thus, our work presents the first human TRIM family member that recognizes HIV-1 capsid and accelerates its uncoating.

Multiple HIV-1 capsid binding proteins have been identified [15, 16, 25, 27–30], most of which, like CSPF6, NUP153, NUP358, TNPO3 and CypA, were shown as dependency factors for HIV-1 replication [25, 28–30]. These factors aid properly uncoating of HIV-1 capsid or escorting viral DNA into nucleus. In the other side, some capsid-binding proteins could perturb uncoating and impede HIV-1 replication. MxB was identified as an IFN-β inducible restriction factor that could block viral DNA nuclear entry by interacting with HIV-1 capsid to increase its stability [16]. In this study, we introduced TRIM11 as a new HIV-1 capsid binding factor that will tilt the balance of uncoating process in favor of accelerating when it is overexpressed in cells, which will result in reduced viral reverse transcription levels.

TRIM11 is not the only human TRIM family member that could associate with HIV-1 capsid. TRIM6 and TRIM34, which are closely related to TRIM5α in sequence similarity, have been reported to bind in vitro assembled HIV-1 capsid, but they do not have the ability to restrict HIV-1 replication [20]. By analysis of extensive TRIM5α_rh_ mutants, Yang et al., demonstrated that binding to HIV-1 capsid is necessary, but not sufficient, for HIV-1 restriction [31]. Cytoplasmic body formation and E3 ubiquitin ligase activity were also implicated in TRIM5α_rh_-mediated capsid disruption and restriction of reverse transcription [32–34]. The restriction activity of TRIM5α was recently reported to correlate with its ability to induce TAK-1 dependent innate immune signaling [35]. Thus, in addition to binding with capsid, some domains of TRIM5α_rh_ must function in a cryptic mechanism to accelerate viral capsid uncoating. In accordance to these findings, the chimeric proteins TRIM-CypA and Trim-NUP153(C), which use different domain for binding to capsid, sustain the restriction ability of TRIM5α_rh_ [36–38]. Then other than binding to HIV-1 capsid, how TRIM11 exerts the function of accelerating uncoating is still unknown.

We previously reported that TRIM11 decreased HIV-1 reverse transcription in a proteasome independent manner [18]. In accordance with this result, here we found that the effect of TRIM11 on viral uncoating is independent of proteasome. Our findings suggested that the mechanism by which TRIM11 accelerates HIV-1 uncoating is not exactly the same as that of TRIM5α_rh_ which can be blocked by MG132 [4, 5]. Furthermore, premature disassembly of HIV-1 imposed by TRIM11 is also independent of the lysosomal pathway. In contrast, microtubule dynamics is essential for TRIM11-mediated uncoating and reduction of reverse transcription. Since microtubule dynamics are involved in uncoating [22], we speculate that this cellular apparatus, that originally supports early stages of HIV-1 replication, may be utilized by antiviral proteins like TRIM5α_rh_ and human TRIM11 to accelerate uncoating.

We compared the effects of TRIM11 and TRIM5α_rh_ on viral uncoating, reverse transcription and transduction, and found that although they have comparable effects on viral reverse transcription, TRIM11 restricts HIV-1 transduction less potently than TRIM5α_rh_ does. We attribute this to the fact that TRIM11 lacks ability to impede the accumulation of nuclear viral DNA. In addition, HIV-1 reverse transcription levels were significantly increased and the viral capsid was more stable in TRIM11 knockdown THP-1-derived macrophages, suggesting that physiological levels of TRIM11 in HIV-1 relevant cells impede viral reverse transcription by accelerating uncoating process. Although TRIM11 expression was shown negatively correlated with HIV-1 replication in monocytes [39], it seems like TRIM11 does not act as a restriction factor like TRIM5α_rh_ that can be a barrier for retrovirus cross species transmission. Unlike Rhesus macaques, human is the natural host of HIV-1, which means the virus has evolved well enough to conquer potential obstacles in the host. We previously reported that HIV-1 Vpr regulated TRIM11 expression levels bidirectionally [18]. However, whether the regulation of TRIM11 by Vpr is a strategy used by HIV-1 to overcome the restriction effect of TRIM11 remained unclear. We speculate that this regulation by Vpr is more relevant to the regulation of innate immune signaling, as Vpr has not been reported to be involved in HIV-1 uncoating. Another scenario is that maybe the restriction of TRIM11 on HIV-1 replication is covered by other cellular factors. Thus, changing TRIM11 expression levels by genetic means trumps the effect of other cellular factors, and evidences its influence on HIV-1 uncoating process. Further investigation into the mechanism by which HIV-1 escapes TRIM11-mediated restriction of early replication is needed.

## Conclusion

Our data demonstrate that human TRIM11 not only has the ability to bind in vitro assembled HIV-1 CA-NC complexes, but also associates with HIV-1 capsid protein during virus replication. In addition, we found that, similar to rhesus macaque TRIM5α, the capacity of TRIM11 to accelerate HIV-1 uncoating and decrease viral reverse transcription is dependent on microtubule dynamics. Finally, our results showed that in one of HIV-1 target cell, THP-1-derived macrophages, TRIM11 exhibits the same ability to interfere with early stages of HIV-1 replication as it does in HEK293 cells. Our work presents the first human TRIM family member that accelerates HIV-1 uncoating by interacting with the capsid, and leads to inhibition of HIV-1 reverse transcription.

## Methods

### Cell culture, reagents and antibodies

HEK293 cells and HEK293T cells were cultured in Dulbecco’s modified Eagle medium (DMEM, Gibco) containing 10 % fetal bovine serum (FBS, Gibco) and 1 % glutamine (Gibco). THP-1 cells and Jurkat cells were cultured in Roswell Park Memorial Institute medium (RPMI, Gibco) supplemented with 10 % FBS and 1 % glutamine. The cells were cultured at 37 °C with 5 % CO_2_.

The antibodies and reagents used in this work were as follows: anti-HA (Cell signaling technology,CST), anti-p24 (Santa Cruz Biotechnology), anti-TRIM11 (Sigma-Aldrich), anti-Myc (Roche); puromycin and MG132 (Sigma-Aldrich), chloroquine (CST), nocodazole (Beoytime) and taxol (Tocris Bioscience).

### Generation of stable cell lines

C-terminal HA-tagged TRIM5α_hu_ and TRIM5α_rh_ were obtained by PCR using cDNA from HEK293 and RhFK-4 cells respectively. Then stably overexpressing cell lines were constructed as previously described [18]. For the TRIM11 stable knockdown cell lines, the shRNA were constructed into pLKO.1 (Ctrl: TTCTCCGAACGTGTCACGT, 1:TATTCATCTTTCCCGAGAT, 2:CTATTACAATTCCTCGGAA). HEK293T cells were co-transfected with pLKO.1, psPAX2 and pMD2.G using Fugene HD Transfection Regent (Promega). Lentiviruses were collected at 48 h after transfection, filtered through 0.45 μm filters. HEK293 cells or THP-1 cells were infected with the lentivirus, and 24 h later infected cells were selected in medium containing 1.1 μg/ml puromycin. For THP-1 cells, the selective concentration of puromycin was 2.7 μg/ml.

### Generation of HIV-1 CA mutations

Point mutations in the CA region of NL4-3 were engineered by oligonucleotide-directed single-round PCR. Specifically, primers containing the mutations were applied for PCR, using pNL4-3.Luc.R-E-plasmid as template and KOD-Plus-Neo (TOYOBO) as DNA polymerase. The PCR products were subjected to DpnI (New England Biolab) at 37 °C for 4 h, and self-ligated for transfection into *E. coli.* The CA region mutant clones were sequenced for confirmation.

### Western blotting

HEK293 cells were collected and lysed with cell lysis buffer (Beyotime) for 10 min on ice. After centrifuged at 12,000×*g*, 4 °C for 10 min, the supernatants were added to 5 × SDS loading buffer (Beyotime) and incubated at 95 °C for 10 min. Samples were loaded onto 10 % SDS-PAGE. The protein concentrations were detected using BCA Protein Assay kit (Beyotime). After separation, the proteins were transferred to nitrocellulose membranes (Bio-rad) and detected by incubation with HRP-conjugated antibodies.

### Single-cycle infectivity assay

The HIV-1 pNL4-3.Luc.R-E-plasmid [40], was cotransfected with pVSVG using X-treme HP (Roche) to produce Vpr-negative pseudotyped HIV-1 NL4-3.Luc viruses (HIV-1). The MLV pSra-luc plasmid, which was provided by Dr. Guangxia Gao (Institute of Biophysics, Chinese Academy of Science), was cotransfected with phit60 and pVSVG using X-treme HP. Viruses were collected at 48 and 72 h after transfection. The collected virus was centrifuged for 30 min at 4500×*g*, and filtered through 0.45 μm filters. The quantity of HIV-1 was assessed with HIV-1 p24 ELISA kit (Advanced BioScience Laboratories). HEK293 cells stably expressing TRIM-HA or knocking down of TRIM11 were seeded in 12-well plates, and infected with different amounts of HIV-1 for 24 h or MLV for 48 h. THP-1 cells stably expressing TRIM11-HA or knocking down of TRIM11 were seeded in 12-well plates in the presence of 100 nM PMA for 48 h, followed by 48 h inoculation with different amounts of HIV-1. Cells were collected and luciferase activity was measured using the Luciferase Assay System (Promega). The luciferase activities were normalized to the protein quantity measured by BCA Protein Assay kit.

### Relative real time PCR

HEK293 cells stably expressing TRIM-HA proteins or knocking down of TRIM11 were seeded in 12-well plates, and infected with HIV-1 pretreated with 100 U/ml DNaseI (TaKaRa) for 30 min at 37 °C. Cells were collected at the indicated times after infection. Genomic DNA was extracted with DNA extraction kit (Tiangen). Real-time PCR was performed as described previously [18] using SYBR Select Master Mix (Thermo Fisher scientific) with primers for reverse transcripts specific for luciferase (Luc-F: AAAAGTTGCGCGGAGGAGTT, Luc-R: ATTTGGACTTTCCGCCCTTCT) and GAPDH (F: AAGGCTGTGGGCAAGG, R: TGGAGGAGTGGGTGTCG).

### The fate-of-capsid assay

The fate-of-capsid assay was performed as previously described [3]. HEK293 cells were seeded in two 10 cm^2^ dishes. After the cell density reached 80 %, the cells were incubated with HIV-1 pseudotyped viruses with or without VSV-g package for 30 min at 4 °C, and shifted to 37 °C for the indicated time. THP-1 cells were seeded in four 10 cm^2^ dishes in the presence of 100 nM PMA for 48 h before infected with HIV-1. The cells were washed with ice-cold PBS for three times, and resuspended with ice-cold PBS containing 1 mM EDTA, and centrifuged at 500×*g* 4 °C for 7 min. The pellets were lysed with 2.65 mL hypotonic lysis buffer (10 mM Tris–HCl pH 8.0, 10 mM KCl, 1 mM EDTA and Complete protease inhibitor) on ice for 15 min. After centrifugation at 1000×*g* 4 °C for 4 min, the supernatants were layered onto 9.7 mL 50 % sucrose cushion (wt/vol, made in PBS), and ultracentrifuged at 120,000×*g* 4 °C for 2 h in a Beckman SW41 rotor. After centrifugation, the pellets were resuspended in 1 × SDS loading buffer (Bio-rad) from bottom of the tubes. The cell lysates before ultracentrifugation were taken as input. The cell lysates and pellets were subjected to Western blotting with anti-HIV-1-p24 antibody.

### In vitro capsid binding assay

HIV-1 CA-NC and TRIM11 encoding sequence was constructed into the pGEX-6P-1 vector. After transformation into *E. coli.* BL21, HIV-1 CA-NC and TRIM11 were expressed and purified by a GST affinity column chromatography with glutathione sepharose4B (GE healthcare). Proteins were cleaved from GST using PreScission Protease (GE healthcare). The CA-NC protein was assembled in vitro by mixed with DNA oligo-TG50 in assemble buffer (50 mM Tris–HCl, pH 8.0, 0.5 mM NaCl). The mixture was incubated at 4 °C overnight and centrifuged at 8600×*g* for 5 min. The cells expressing TRIM11-HA and TRIM5α_hu_-HA were lysed with hypotonic lysis buffer for 30 min on ice and precipitated using Monoclonal Anti-HA-Agarose antibody (Sigma-Aldrich). The cells expressing TRIM5α_rh_-HA were lysed with hypotonic lysis buffer for 15 min on ice and centrifuged at 12,000×*g* for 10 min. The cell lysate and HA-precipitated mixture were incubated with CA-NC protein for 1 h at room temperature. For the purified TRIM11 protein, 8 μg proteins were used to incubate with in vitro assembled CA-NC complexes. The mixtures were layered onto 70 % sucrose cushion (wt/vol, made in PBS), and ultracentrifuged at 100,000×*g* 4 °C for 1 h in a Beckman SW60 rotor. After centrifugation, the pellets were resuspended in 1 × SDS loading buffer from bottom of the tubes. The cell lysates and pellets were subjected to Western blotting with anti-HIV-1-p24 antibody and anti-HA antibody. The cell lysates before ultracentrifugation were taken as input.

### PLA assays

The PLA detection was carried out using the Duolink in situ PLA kit (Sigma-Aldrich) according to the instructions of the manufacturer. Briefly, cells were fixed by fixative solution (Beoytime) followed by blocking using the Duolink blocking solution (3 drop) at room temperature for 1 h. Primary antibodies were added at dilution of 1:100 (anti-HA) and 1:20 (anti-p24) in 40 μl PBS with 3 % BSA and 0.3 % Triton X-100 and incubated at 37 °C for 1 h. The slides were washed two times with PBS for 5 min each. After incubation with the secondary antibodies, ligation, amplification and mounting according to the instructions, images were acquired using a Nikon A1 MP two-photon microscope.

### Statistical analyses

The data are presented as the mean ± SD of three independent experiments that were performed in triplicate, and all data were analyzed using Student’s *t* test. P < 0.05 (*) was considered to be statistically significant.

## Additional file

10.1186/s12977-016-0306-5 TRIM11 expression levels in different cells. HEK293, THP-1, PMA treated THP-1 cells and Jurkat cells were lysed with cell lysis buffer. After centrifugation, the supernatants were subjected to western blotting for detection of TRIM11 expression levels.
