# Supplementary figures and images for: An HIV-1 capsid binding protein TRIM11 accelerates viral uncoating
Source: Retrovirology. 2016 Oct 13;13:72. doi: 10.1186/s12977-016-0306-5 (PMC5062926; doi:10.1186/s12977-016-0306-5)

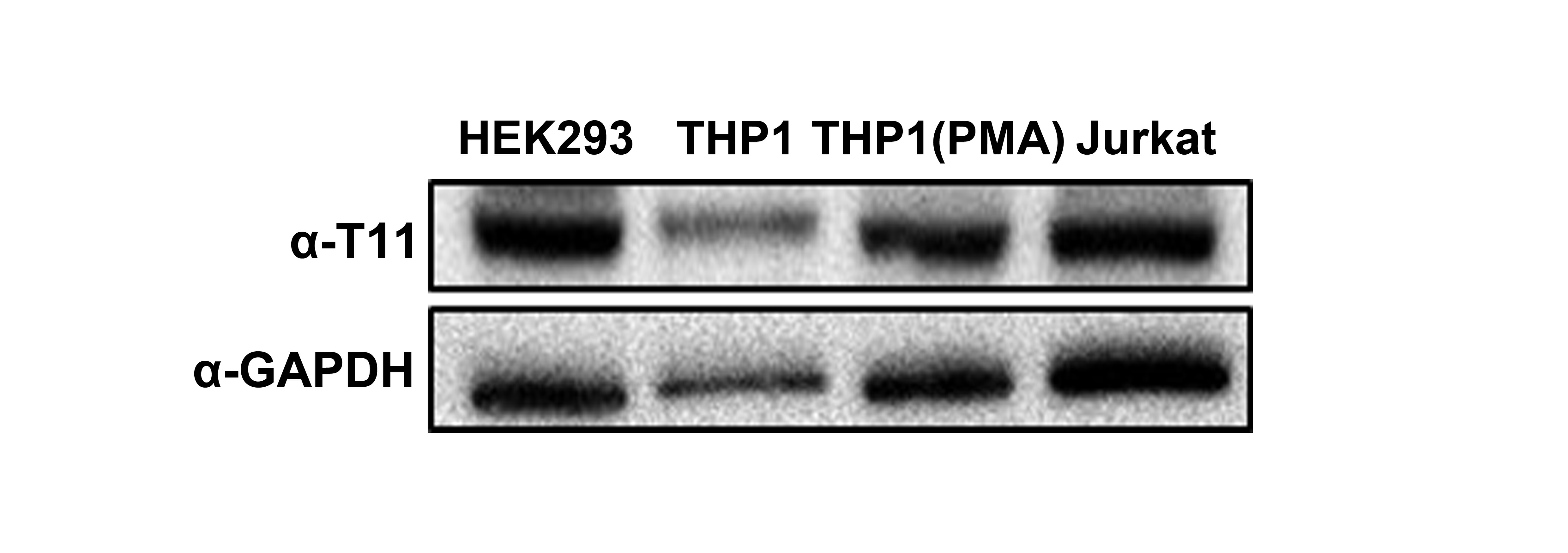

Supplement: Supplementary file 1 — 10.1186/s12977-016-0306-5 TRIM11 expression levels in different cells. HEK293, THP-1, PMA treated THP-1 cells and Jurkat cells were lysed with cell lysis buffer. After centrifugation, the supernatants were subjected to western blotting for detection of TRIM11 expression levels. [file 12977_2016_306_MOESM1_ESM.tif]
